# Application of machine learning based genome sequence analysis in pathogen identification

**DOI:** 10.3389/fmicb.2024.1474078

**Published:** 2024-10-02

**Authors:** Yunqiu Gao, Min Liu

**Affiliations:** ^1^Department of Dermatology, The First Hospital of China Medical University, Shenyang, China; ^2^Key Laboratory of Immunodermatology, Ministry of Education and NHC, National Joint Engineering Research Center for Theranostics of Immunological Skin Diseases, Shenyang, China; ^3^Institute of Respiratory Disease, China Medical University, Shenyang, China

**Keywords:** artificial intelligence (AI), antibiotic resistance, pathogenic microorganisms, machine learning (ML), diagnosis

## Abstract

Infectious diseases caused by pathogenic microorganisms pose a serious threat to human health. Despite advances in molecular biology, genetics, computation, and medicinal chemistry, infectious diseases remain a significant public health concern. Addressing the challenges posed by pathogen outbreaks, pandemics, and antimicrobial resistance requires concerted interdisciplinary efforts. With the development of computer technology and the continuous exploration of artificial intelligence(AI)applications in the biomedical field, the automatic morphological recognition and image processing of microbial images under microscopes have advanced rapidly. The research team of Institute of Microbiology, Chinese Academy of Sciences has developed a single cell microbial identification technology combining Raman spectroscopy and artificial intelligence. Through laser Raman acquisition system and convolutional neural network analysis, the average accuracy rate of 95.64% has been achieved, and the identification can be completed in only 5 min. These technologies have shown substantial advantages in the visible morphological detection of pathogenic microorganisms, expanding anti-infective drug discovery, enhancing our understanding of infection biology, and accelerating the development of diagnostics. In this review, we discuss the application of AI-based machine learning in image analysis, genome sequencing data analysis, and natural language processing (NLP) for pathogen identification, highlighting the significant role of artificial intelligence in pathogen diagnosis. AI can improve the accuracy and efficiency of diagnosis, promote early detection and personalized treatment, and enhance public health safety.

## Introduction

Pathogenic microorganisms include viruses, bacteria, parasites, and fungi that can cause infections in humans and animals. They spread rapidly through aerosols, body fluids, food, and direct contact, leading to various infectious diseases and even death (Zhang et al., 2018). Early detection, diagnosis, and treatment are crucial for preventing infectious diseases. Since the discovery of penicillin in 1928, antibiotics have become vital public health tools, saving countless lives globally (Fleming, 2001; Davies and Davies, 2010). Today, a wide range of antibacterial, antifungal, and antiviral drugs are used in clinical practice. However, the misuse of these antimicrobial drugs has led to increased drug resistance in microorganisms, reducing the effectiveness of these treatments, a phenomenon known as antimicrobial resistance (Prestinaci et al., 2015). According to the World Health Organization (WHO) in 2020, antimicrobial resistance (AMR) is among the top ten global public health threats facing humanity. In 2022, The Lancet published a systematic analysis of the global burden of bacterial AMR, including data from over 200 countries. The study revealed that AMR poses a significant threat to global health. In 2019, AMR infections directly caused approximately 1.27 million deaths and indirectly resulted in about 4.95 million deaths worldwide (Antimicrobial Resistance Collaborators, 2022). By 2022, around 1.3 million deaths were related to antibiotic resistance (Ranjbar and Alam, 2023). If left unaddressed, it is projected that by 2050, antibiotic-resistant infections could cause 10 million deaths annually, with direct economic losses exceeding $10 trillion (de Kraker et al., 2016; Ventola, 2015). Developing new antimicrobial drugs is becoming increasingly difficult, often taking 10–15 years and costing over 6 billion (Wouters et al., 2020; DiMasi et al., 2016). The emergence of more severe multidrug-resistant bacteria will pose significant treatment challenges. These data highlight the substantial burden that infectious diseases and antimicrobial resistance place on human health and the global economy.

**Figure 1:**
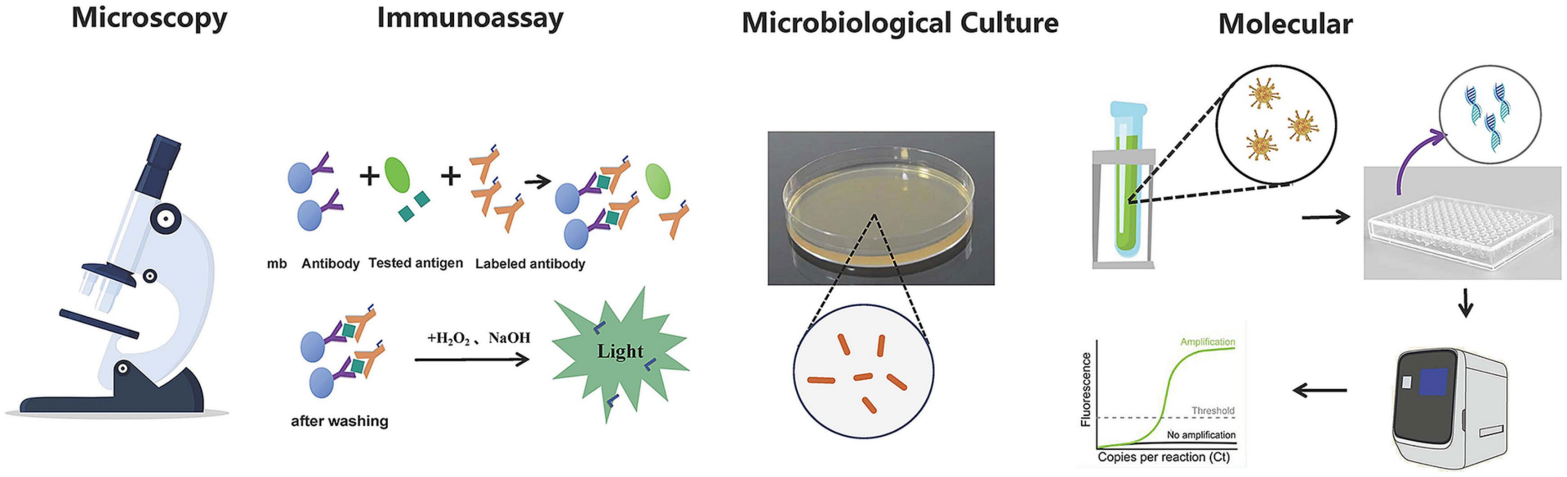
Pathogenic microorganism detection technology.

The technologies in pathogen detection include nucleic acid and immunological methods (Whiley and Taylor, 2016) (Figure 1). These technologies help identify pathogenic bacteria or potential health risks, making accurate and rapid detection crucial for diagnosing and preventing diseases in public health, environmental pollution monitoring (Zhang et al., 2023), and clinical diagnosis (Smith and Kirby, 2020). However, current detection techniques often fall short of clinical needs due to long processing times, cumbersome procedures, and reliance on large instruments, limiting fast and efficient identification. The traditional methods for identifying pathogenic microorganisms, including smear microscopy, isolation and cultivation, biochemical assays etc., are not without limitations. These methods are often characterized by prolonged timeframes, intricate procedures, and suboptimal sensitivity. A case in point is the identification of mycobacterial strains, which can extend to a lengthy period of 30 to 40 days. Furthermore, certain fastidious bacteria and viruses demand cultivation conditions that are so stringent they may prove unattainable, or the organisms may be refractory to culture altogether. Molecular diagnostic techniques, anchored in PCR, have made strides in addressing some of the aforementioned challenges in pathogen detection. However, they encounter significant hurdles when it comes to the identification of unknown microorganisms. The absence of known nucleic acid sequences renders the design of specific primers an insurmountable obstacle for these technologies. While immunological and PCR methods boast high sensitivity and specificity, enabling the detection of a broad spectrum of pathogens, they are constrained by their targeted nature. This means that a single experiment is typically capable of detecting only one pathogen, which can lead to diminished diagnostic efficiency. The indistinguishable symptoms and signs of many infectious diseases further complicate matters, as identical clinical presentations may be induced by a variety of pathogens or result from co-infections. The laborious and time-consuming process of detecting pathogens one at a time can potentially lead to diagnostic delays.

A key breakthrough in overcoming these limitations is the deployment of AI driven genome sequencing tools, which analyze complex genomic data to quickly and accurately identify pathogenic microorganisms with high throughput and speed. For example, DeepVariant is a mutation caller based on deep learning that can improve the accuracy score of single nucleotide mutation and Indel detection (Poplin et al., 2018). Integrating image processing and big data analysis into detection methods is therefore highly significant (Kothari et al., 2014; Jain et al., 2016). Recent advancements in AI, particularly in computer vision and image processing, have shown promising potential in the morphological detection of pathogenic microorganisms.

**Figure 2:**
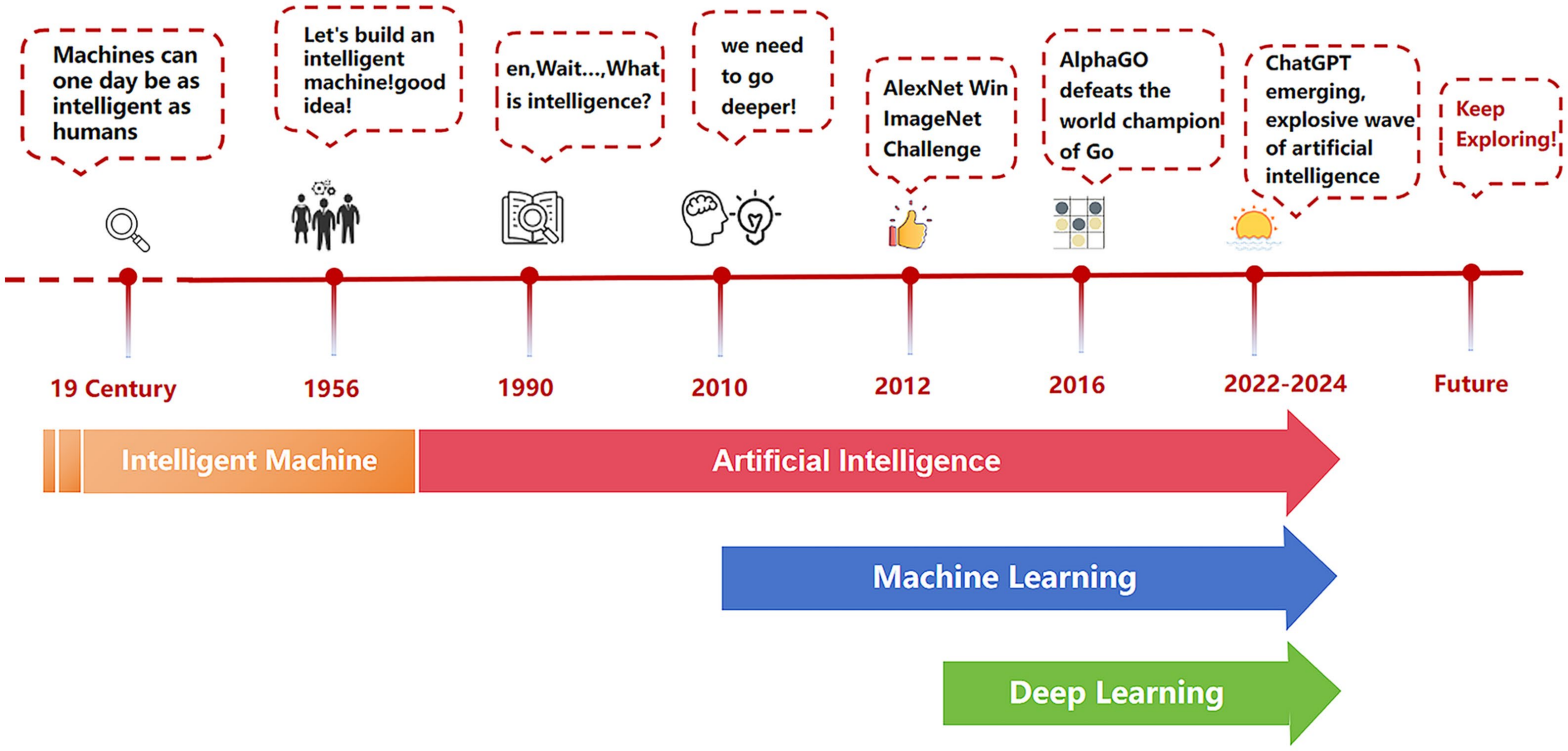
Major milestones in the development of artificial intelligence.

The development of AI has progressed through several key stages. It began in 1945 with Alan Turing’s idea of using computers to simulate the human brain. During the 1950s to the 1970s, AI started to become practical with the creation of the first generation of AI systems. The 2010s saw an explosion in AI capabilities, driven by advances in deep learning and big data technologies like chatGPT (LeCun et al., 2015; Esteva et al., 2017). Today, AI excels in numerous fields, including disease diagnosis, risk management, facial recognition (Figure 2).

AI has significant applications in microbial diagnosis. It uses machine learning algorithms to analyze microbial genome data, identify antibiotic resistance genes, speed up pathogen identification, and improve diagnostic accuracy. AI can also process vast amounts of complex data, provide real-time diagnostic support, aid in the early detection and control of infectious diseases, and enhance public health prevention and control efforts (Esteva et al., 2017).

The potential of AI in microbiology is yet to be fully realized. Microbial research generates vast amounts of biological image data, and AI has proven crucial in analyzing high-throughput sequencing data and using natural language processing to identify pathogenic microorganisms. Traditional computing methods are slow in processing these data, whereas AI, especially deep learning, excels in both accuracy and speed (Camacho et al., 2018; Ching et al., 2018). Deep learning has introduced new applications to microbial research, significantly advancing microbial identification and diagnosis. The application of deep learning in microbial image recognition and classification has grown rapidly (Wainberg et al., 2018; Cao et al., 2018; Jiang et al., 2022). This article reviews the use of AI in identifying and diagnosing pathogenic microorganisms.

## Application of AI in image analysis of pathogenic microorganisms

**Figure 3:**
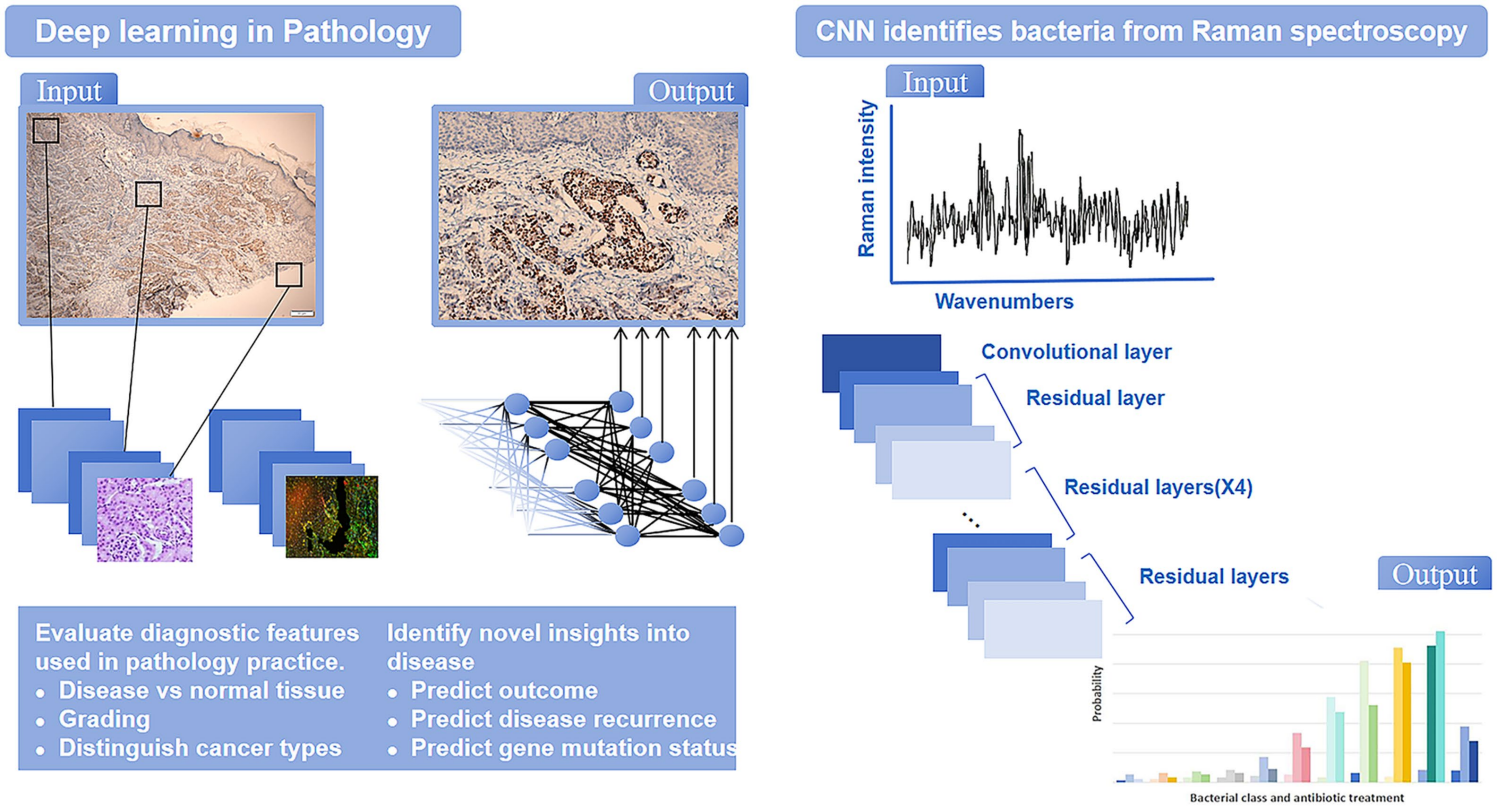
The application of AI related algorithms in image analysis.

AI, particularly machine learning and deep learning, has made significant strides in the automatic recognition and classification of pathogenic microorganisms in microscope images. These technologies effectively analyze and classify bacteria, viruses, fungi, and parasites. Deep learning has made microscope image analysis more efficient and universal, enabling accurate cell detection and classification (Figure 3). Compared to traditional methods, deep learning significantly enhances the accuracy and reliability of microorganism detection (Esteva et al., 2021; Chen and Asch, 2017).

To address the challenges of pathogen detection, particularly with large sample sizes and the identification of difficult bacteria, researchers have been exploring intelligent clinical microbial morphology testing. In 2020, Professor Aydogan Ozcan’s team at the University of California developed a highly sensitive, precise, timely, and low-cost microbial online monitoring AI platform. This system combines coherent microscopy imaging with deep neural network analysis to enable the intelligent identification and classification of live microorganisms. By analyzing growth delay holograms, the system achieves rapid detection of bacterial growth and species classification, with a detection limit of approximately 1 CFU/L for *Escherichia coli*, *Klebsiella pneumoniae*, and *Pseudomonas aeruginosa* within ≤9 h. This significantly reduces the testing time compared to the EPA gold standard method, which takes at least 24 h (Wang et al., 2020). Similarly, a team from the University of Geneva in Switzerland has developed an automated urine culture analysis system. The WASPLab software automatically reads and analyzes bacterial colony images on urine culture plates, quickly reporting urine culture results. Using automated equipment, the turnaround time is reduced by nearly 50%, minimizing manual reading errors and improving detection efficiency and accuracy (Cherkaoui et al., 2020).

Currently, the interpretation of imaging results relies heavily on the subjective clinical experience of professional imaging doctors. Clinically, there is a strong expectation for the testing department to diagnose pathogens rapidly and provide accurate drug sensitivity results. AI is now widely used in medical imaging, particularly in detecting and diagnosing infectious diseases. For instance, during the global COVID-19 outbreak in 2019, AI significantly improved the diagnostic accuracy and efficiency of chest CT scans and X-rays, enabling rapid and precise screening, identification, and characterization of COVID-19 (Hassan et al., 2022). AI also aids in detecting and analyzing secondary pulmonary infections in COVID-19 patients, enhancing diagnostic accuracy and helping to assess disease severity and predict clinical outcomes (Viswanathan et al., 2022). In lymphoma patients, deep learning accurately identifies high metabolic tumor sites in 18F-FDG-PET/CT scans, potentially aiding in excluding metabolically active diseases (Ikeda et al., 1987). These studies highlight AI’s potential in enhancing diagnostic efficiency and accuracy for infectious diseases and its broad application prospects in medical imaging.

**Figure 4:**
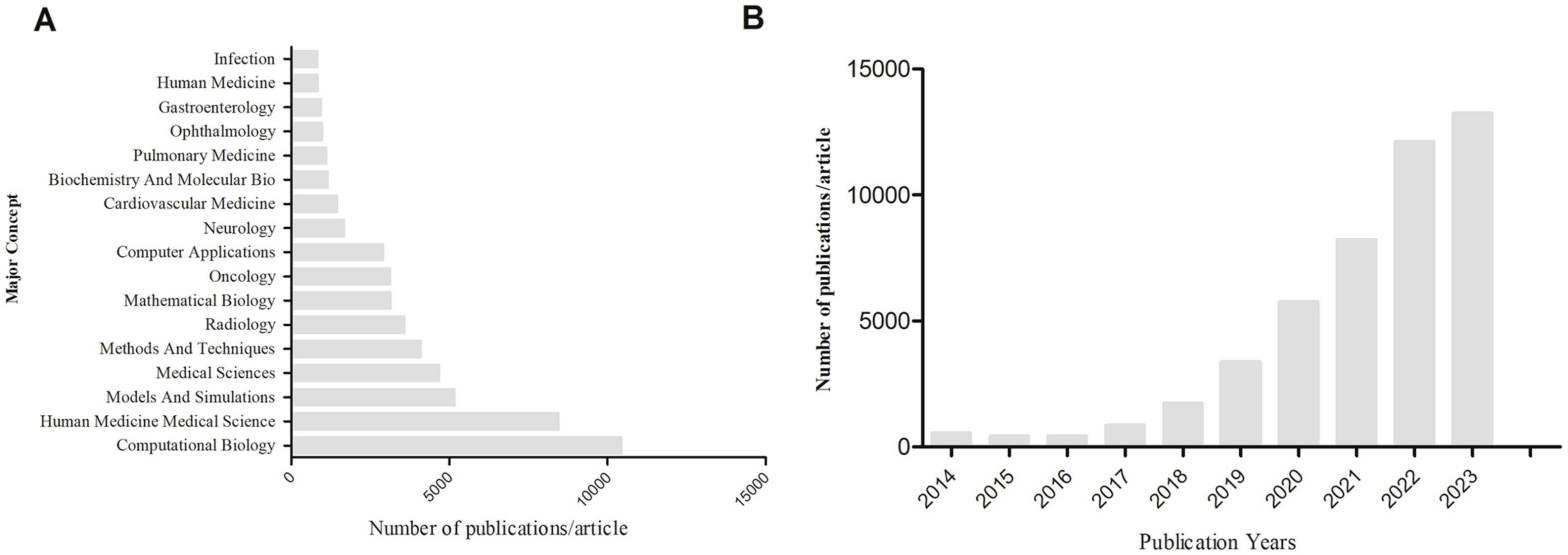
**(A)** Search for keywords “artificial intelligence” and “medical imaging” to rank the top major concepts in terms of article volume. **(B)** The number of articles and publications retrieved using “artificial intelligence” and “medical imaging” as keywords from 2014 to 2023.

We conducted a bibliometric analysis using the Web of Science database to search for original research on the application of AI in medical imaging over the past decade, with the keywords “Artificial Intelligence” and “Medical Imaging.” We analyzed the retrieved literature and generated a citation report. A total of 50,547 articles were found, with a notable increase in publication volume since 2020. Europe and the United States remain leaders in this field, while Chinese scholars have shown rapid development in the past 2 years, now leading in publication volume. However, the impact of Chinese research is relatively low, indicating an academic quality gap with European and American countries in AI-assisted medical imaging (Tables 1, 2; Figure 4). Most clinical research focus on using deep learning and its derivative algorithms to improve image segmentation accuracy and assist clinical diagnosis. According to our statistical results, AI ranks ninth in the field of infection research. With significant progress in AI-driven microbial microscopy image detection, the application of deep learning in microbial image recognition and classification has immense development potential.

**Table 1:** Top 10 countries/regions medical imaging in artificial intelligence research from 2014 to 2023.

Rank	Countries/regions	Number of publications/article
1	CHINA	14,338
2	USA	11,309
3	INDIA	5,377
4	ENGLAND	3,291
5	UK	3,184
6	SOUTH KOREA	2,575
7	GERMANY	2,505
8	CANADA	2,390
9	SAUDI ARABIA	1946
10	ITALY	1932

**Table 2:** Top 10 research area in artificial intelligence research from 2014 to 2023.

Rank	Research area	Number of publications/article
1	Mathematical Computational Biology	42,691
2	Radiology Nuclear Medicine Medical Imaging	36,960
3	Engineering	35,466
4	Communication	33,268
5	Mathematics	20,565
6	Neurosciences Neurology	9,151
7	Science Technology Other Topics	9,003
8	Imaging Science Photographic Technology	6,757
9	Oncology	6,608
10	Automation Control Systems	6,347

## The application of AI in genome sequencing data analysis

AI is widely used in analyzing next-generation sequencing (NGS) data, particularly for pathogen identification and classification. AI technology can quickly process NGS data and identify pathogens in samples, which is crucial for the timely diagnosis of infectious diseases. In a study published in Nature Medicine, scientists have developed an AI framework that integrates a multi detection platform for detecting and identifying biomolecules. The system analyzes three representative plasmids with different color signals, which are derived from drug-resistant *Klebsiella pneumoniae* bacteria. Compared with traditional technology, this system demonstrates excellent recall and accuracy, detecting 93.8% of events in real-time and achieving a classification accuracy of 99.8%. This study demonstrates the potential of AI in medical diagnosis, especially in clinical environments that require rapid and accurate analysis (Ganjalizadeh et al., 2023). A study published in Scientific Reports, researchers used AI algorithms combined with NGS data from T cell receptors (TCRs) to diagnose glioma patients. This study explores multidimensional classification and feature selection of TCR sequence diversity index, as well as two-dimensional classification and feature selection analysis of TCR related sequences. The results indicate that through these analyzes, researchers were able to identify two sets of core sequences, each containing three sequences, sufficient to achieve a 96.7% accuracy in glioma detection and diagnosis (Zhou et al., 2024).

The metagenomic high-throughput sequencing technology (mNGS) has shown great potential in pathogen detection. It identifies pathogenic microorganisms by directly sequencing nucleic acids in samples, without the need to pre-set target sequences, thus overcoming the limitations of traditional microbial detection methods. The IDseq platform is a cloud based open-source platform developed by the Chan Zuckerberg Initiative. Based on pathogen metagenomics detection technology, high-throughput sequencing technology is used to analyze microorganisms and host nucleic acids in clinical samples, enabling unbiased detection of various pathogenic microorganisms, including bacteria, fungi, viruses, and parasites. This technology has shown important application value in the detection of infectious diseases pathogens, especially when the traditional etiological diagnosis methods are difficult to meet the clinical needs. This platform has the comprehensiveness to process diverse samples and detect numerous pathogens, high sensitivity to improve pathogen detection sensitivity, and in-depth analysis capabilities for drug resistance and virulence analysis. Its open-source nature and cloud computing foundation make it easy to access and process big data on a global scale, reducing the need for bioinformatics experts and local server level hardware resources through automated processes, thereby lowering costs and time. The platform is user-friendly and supports real-time pathogen detection, including newly emerging pathogens. It also supports the generation of environmental background models and data sharing, promoting scientific research collaboration (Kalantar et al., 2020). In a case of pathogen discovery in childhood meningitis in Bangladesh, researchers used the IDseq platform to reanalyze three meningitis samples with the aim of exploring unknown pathogens. These three samples include one meningitis sample caused by *Streptococcus pneumoniae* (CHRF 0002), one meningitis sample caused by chikungunya virus (CHRF 0094), and one water control sample (CHRF 0000). The IDseq platform has successfully identified pathogens through effective host sequence filtering and quality control. Especially in the CHRF 0094 sample, after host filtering and QC steps, the chikungunya virus accounted for 63% of non-host reads, and through the coverage visualization tool of the IDseq portal, researchers were able to observe the whole genome coverage of the chikungunya virus in the sample. This indicates that the IDseq platform can effectively assist researchers in quickly obtaining in-depth insights into sample quality, microbial content, and cohort trends (Saha et al., 2019).

AI algorithms can accurately classify pathogens based on genomic data, which is crucial for monitoring their evolution and transmission. MetaPhlAn (Metagenomic Phylogenetic Analysis) is a widely used bioinformatics tool that provides species-level analysis of microbial composition from metagenomic shotgun sequencing data. A 2023 Nature article detailed how researchers integrated extensive new microbial genome and metagenomic data into the MetaPhlAn database, defining 26,970 Species-Level Genome Bins (SGBs). This expansion allows MetaPhlAn 4 to analyze metagenomic data more accurately, particularly in identifying uncharacterized species and improving the explanatory power of microbial community composition analysis (Blanco-Miguez et al., 2023). Antibiotic resistance is a pressing global health threat. Rapid whole-genome sequencing offers opportunities to predict antibiotic resistance from genomic data. In 2024, the Helmholtz Center for Infection Research in Braunschweig, Germany, evaluated four advanced machine learning methods (Kofer, PhenotypeSeeker, Seq2Geno2Pheno, and Aytan Aktig), a baseline ML method, and ResFinder. The results showed significant performance differences among these technologies and datasets, with ML methods excelling in closely related strains and ResFinder performing better with more divergent genomes. ResFinder, combining AI technology, can detect and classify antibiotic resistance genes from NGS data, providing crucial data for public health monitoring (Hu et al., 2024).

To address data diversity, break down information silos, meet the demands of big data analysis, enhance research efficiency, support interdisciplinary research, and leverage modern information technology, integrating databases and knowledge bases has become essential. AI algorithms, combined with extensive databases like NCBI^1^ and EMBL-EBI^2^, and knowledge bases, can significantly improve the accuracy of pathogen identification and classification. For instance, Kraken2 is a highly efficient pathogen classification tool that uses AI technology and a comprehensive reference database to enable rapid analysis of NGS data.

## Application of NLP in identification of pathogenic microorganisms

What is Natural Language Processing (NLP)? NLP is a machine learning technology that enables computers to interpret, process, and understand human language. It serves as a crucial bridge for communication between humans and machines.

Medical literature is an essential resource for both medical and clinical research. The vast variety of pathogenic microorganisms and parasites associated with infectious diseases, however, poses significant challenges for doctors and researchers when it comes to consulting and organizing this massive volume of literature. The application of NLP technology facilitates the extraction of valuable insights from medical literature and enhances the accuracy and convenience of laboratory data analysis. NLP technology can process microbial data through structured data techniques, such as standardizing EMR (Electronic Medical Records) and laboratory data, then storing this information in databases. Additionally, deep learning algorithms can denoise, segment, and extract features from imaging data (Ananiadou et al., 2010; Chen et al., 2015; Wang et al., 2018; Lee et al., 2020; Rajkomar et al., 2019). An article published in Scientific Reports in 2024 introduced a MarkerGeneBERT system, an NLP system developed by CapitalBio Technology, which automatically extracts information on species, tissues, cell types, and cell marker genes from single-cell sequencing literature. In a study, the system extracted 8,873 human and 9,064 mouse cell markers from 3,987 studies, demonstrating 76% completeness and 75% accuracy, surpassing the CellMarker2.0 system. In addition, MarkerGeneBERT has discovered 89 new cell types and 183 new marker genes. In terms of gene recognition, the system achieved an F1 score of 87%, with a cell name recognition accuracy of 92%. More than 20,000 genes and 4,000 cell types were identified from literature, with accuracies of 90.8 and 92.7%, respectively. Additionally, 1764 new cell types were added, all of which were not previously recorded in the database (Cheng et al., 2024).

In 2022, David Burstein’s team published an article in Nature Communications on using NLP to interpret microbial gene function. They developed a deep learning model that utilized gene embeddings, calculated based on the co-occurrence rate of gene families, as input for a classifier to predict gene function. The word2vec algorithm was employed to calculate the gene embedding space, providing a simple, fast, and direct method. Through scarcity analysis, the study highlighted functional categories with high discovery potential and uncovered hypothetical bacterial membrane-binding mechanisms and microbial defense systems in the human microbiome. Additionally, NLP models can be fine-tuned to explore specific systems or functions, such as training classifiers for particular genes or creating new embeddings using relevant corpora (such as virus genomes, specific microbial communities). This approach is applicable not only for inferring functions of genes without sequence similarity to characteristic proteins but also for exploring diverse functions of homologous genes. This greatly enhances the understanding of microbial gene functions and aids in interpreting unknown microbial gene functions and evolution (Miller et al., 2022). In the same year, another article in Nature Communications introduced a universal “gene semantic” model using NLP. This model employed convolutional neural networks (CNN) to classify peptide sequences and identify potential antimicrobial peptides (AMPs). The deep learning model demonstrated significantly higher accuracy and recall in identifying AMPs compared to traditional methods. A new set of AMPs sequences was identified from the human gut microbiome, showing strong antibacterial activity *in vitro* and validating the model’s predictions (Ma et al., 2022).

## Representative case

Antibiotics have been used to treat life-threatening infections for nearly a century, but with the increase of drug-resistant bacteria, traditional therapies are no longer effective against these infections. The crisis of antibiotic resistance has become an urgent global health issue that requires the discovery of a new generation of nucleic acid and peptide based antibiotics. However, traditional methods for developing antimicrobial peptides (AMPs) are slow and costly.

In 2023, Nat Commun published an article exploring methods to accelerate the development of AMPs by combining cell-free protein synthesis (CFPS) and deep learning techniques. Researchers use generative deep learning models to learn from a large number of unlabeled natural protein sequences and propose new AMPs sequences. Combined with the CFPS system, this *in vitro* transcription and translation system uses DNA templates for protein synthesis, enabling rapid and small-scale production and screening of hundreds of peptides, overcoming the cytotoxicity issues in traditional cell expression systems. Within 24 h, researchers designed, produced, and screened 500 candidate AMPs, ultimately identifying 30 functional AMPs, of which 6 exhibited high antibacterial activity against multidrug-resistant pathogens and low cytotoxicity to human cells. This study demonstrates the potential of deep learning and CFPS technology in accelerating the development of AMPs, providing an efficient and economical new approach to combat microbial resistance (Pandi et al., 2023).

In 2024, Fudan University and a team of Virtue scientists combined AI and biomedical research to predict nearly 1 million new antimicrobial peptides from the global microbiome. They developed a new machine learning algorithm that effectively reduces the false positive rate in AMP recognition. They predicted nearly 1 million novel non redundant antimicrobial peptides from 63,410 environmental and host related metagenomes worldwide, as well as 87,920 high-quality bacterial and archaeal genomes. They also created the AMP comprehensive database AMPSphere, which was published in the main issue of Cell (Santos-Junior et al., 2024).

In May 2023, Professor James Collins and his team published a paper in Nature Chemical Biology, using AI algorithms to discover a novel antibiotic abaucin that can specifically kill the drug-resistant bacterium *Acinetobacter baumannii*. This study is the first to use AI and interpretable deep learning to discover a groundbreaking new class of antibiotics that are effective against multidrug-resistant pathogens, demonstrating the enormous potential of AI in drug discovery and combating antibiotic resistance (Liu et al., 2023).

In a study published in the journal Antibiotics, researchers used a decision tree based machine learning algorithm to predict antibiotic resistance. This study trained 10 machine learning classifiers and generated predictive models for meropenem, ciprofloxacin, and cefotaxime drugs. Research has found that certain models exhibit higher F1 scores, accuracy, precision, and specificity among all machine learning models used. For example, RandomForestClassifier showed moderate F1 score (0.6), accuracy (0.61), and specificity (0.625) for ciprofloxacin. For cefotaxime, RidgeClassifier performed well and displayed F1 score (0.652), accuracy (0.654), and specificity (0.652) values. For meropenem, KNeighborsClassifier showed moderate F1 scores (0.629), accuracy (0.629), and specificity (0.629) (Yasir et al., 2022). In 2022, a collaboration between the Federal Institute of Technology Zurich, Basel University Hospital, and Basel University used mass spectrometry combined with AI algorithms to identify multidrug-resistant pathogens. Researchers collected over 300,000 clinical strains from four diagnostic laboratories in Switzerland between 2016 and 2018, using Bruker’s MALDI Biotyper microbial mass spectrometry system. The mass spectrometry data were associated with drug resistance information to create the DRIAMS dataset, which includes data for 803 bacterial strains, over 300,000 clinical strains, and 768,300 antibiotic resistance entries for more than 70 antibiotics. Using this dataset, they trained three machine learning algorithms—logistic regression, gradient-boosted decision trees (LightGBM), and deep neural networks (MLP)—to establish a classification model for drug-resistant bacteria. The prediction model was validated with *Staphylococcus aureus*, *Escherichia coli*, and *Klebsiella pneumoniae*, showing AUROC values of 0.80, 0.74, and 0.74, respectively, indicating accurate predictions of antibiotic resistance. This study highlights the significant impact of AI in the image analysis of pathogenic microorganisms. Automated and intelligent image analysis technologies enable medical institutions to diagnose infectious diseases more quickly and accurately, enhancing overall public health prevention and control capabilities (Weis et al., 2022; Tahir et al., 2018).

## Advantages and challenges

The main advantages of AI in diagnosing pathogenic microorganisms are:

1. Rapid Processing and Analysis: AI can quickly process large volumes of microbial data, including genomic and metabolomic information, significantly reducing the time needed for differential diagnosis. AI programs can complete complex data analysis in minutes, saving substantial time compared to traditional methods (Erlich and Narayanan, 2014; He et al., 2010; Topol, 2019).
2. High Accuracy: AI models, through training, achieve high-precision identification and classification, especially with complex microbial communities. Using machine learning and deep learning algorithms, AI can recognize specific microbial features and provide accurate diagnostic results (Knights et al., 2011; Libbrecht and Noble, 2015; Esteva et al., 2017).
3. Automation and Scalability: AI systems automate the microbial identification and diagnosis process, reducing manual operations and improving laboratory efficiency. These models continuously update and optimize with new data, adapting to evolving pathogenic microorganisms (Mamoshina et al., 2016).
4. Data Integration and Knowledge Discovery: AI integrates information from various sources—genomic, metabolite, and clinical data—to offer comprehensive diagnostic insights. Through big data analysis, AI uncovers new characteristics and resistance mechanisms in pathogens, contributing to public health and disease prevention (Marx, 2013; Libbrecht and Noble, 2015; Topol, 2019).

Currently, AI integration in global healthcare is driving a technological revolution. However, AI faces several major challenges:

1. Data Issues: Despite accumulating a large amount of medical data, high-value data is still scarce and scattered. Lack of unified data standards, widespread data silos, and enhanced requirements for personal medical information security (Topol, 2019; Raghupathi and Raghupathi, 2014). The other main challenges faced by AI in processing genomic data include incomplete and noisy data, which may lead to inaccurate analysis results. To overcome these issues, researchers have proposed various strategies, such as using interpolation techniques to fill missing values, using hybrid models to enhance robustness to noise, improving model generalization ability through data augmentation and transfer learning, and applying multi view learning and deep learning techniques to more comprehensively understand and predict genomic data. These methods help improve the accuracy and reliability of genomic data analysis, providing stronger support for researchers and clinical applications (Gupta and Gupta, 2019).
2. Data Interpretability: To prevent errors or inaccuracies in the application of artificial intelligence in healthcare, one can improve the data interpretability of AI models through various strategies. These include the use of transparent and simple algorithms, the application of local and global interpretation techniques, the calculation of SHAP values, the conduct of internal model analyzes, the assurance of model accountability, the inference of causality, the establishment of clear model boundaries, the implementation of adversarial testing, the practice of continuous evaluation, the development of user-friendly interpretations, and the adoption of multimodal interpretation methods. Such methods aid in enhancing user trust in AI decision-making processes, ensuring model transparency and accountability, and fulfilling regulatory requirements (Finlayson et al., 2019; Chu et al., 2023).
3. Data Privacy: In order to protect data privacy in artificial intelligence applications that enhance pathogen identification, various technologies and methods can be adopted, including federated learning, group learning, privacy computing technology, PHDtools platform, and differential privacy. These methods can effectively protect data involving personal privacy while improving the accuracy of pathogen identification by means of collaborative training models, combining edge computing and blockchain, applying homomorphic encryption and secure multi-party computing, developing interactive online platforms, and introducing data processing noise. These developments provide new ideas and solutions for privacy protection of medical data (Obermeyer and Emanuel, 2016; Price and Cohen, 2019; Ahuja, 2019; Martin and Zimmermann, 2024; Khalid et al., 2023).

## Conclusion

With the advancement of algorithmic computing power, computer hardware, and the advent of the big data era, AI technology has flourished and penetrated the medical field, transforming traditional medical practices. This review discusses the significant role of AI in identifying and diagnosing pathogenic microorganisms. Machine learning and deep learning algorithms enable faster, more accurate pathogen recognition with automation, efficiency, high sensitivity, and specificity. AI-assisted imaging technology allows computers to analyze vast amounts of medical imaging data, helping doctors make quicker and more accurate diagnoses. Natural language processing in AI extracts valuable information from scientific literature and databases, aiding clinical decision-making and research. Additionally, AI algorithms accurately classify pathogens based on genomic data, crucial for monitoring pathogen evolution and transmission. Using machine learning to optimize antibiotic use in healthcare settings is a forward-thinking approach to combating antimicrobial resistance now and in the future. In order to further promote the development of this field, interdisciplinary collaboration between artificial intelligence researchers and microbiologists is particularly important. This will help combine the professional knowledge of microbiology with the powerful analytical capabilities of artificial intelligence to jointly develop more accurate and efficient pathogen identification tools.

## Footnotes

^1^ https://www.ncbi.nlm.nih.gov/

^2^ https://www.ebi.ac.uk/
